# Whole‐genome sequencing bulked segregant analysis uncovered *FW7*, a Fusarium wilt resistance gene masked by epistasis in octoploid strawberry

**DOI:** 10.1002/tpg2.70136

**Published:** 2025-10-22

**Authors:** Mishi V. Vachev, Marta Bjornson, Dominique D. A. Pincot, Randi A. Famula, John T. Lovell, Avril M. Harder, Lori H. Handley, Jane Grimwood, Hillel Brukental, Cindy M. Lòpez, Glenn S. Cole, Mitchell J. Feldmann, Steven J. Knapp

**Affiliations:** ^1^ Department of Plant Sciences University of California, Davis Davis California USA; ^2^ Genome Sequencing Center HudsonAlpha Institute for Biotechnology Huntsville Alabama USA; ^3^ Department of Energy Joint Genome Institute California USA

## Abstract

Fusarium wilt, a vascular disease of strawberry (*Fragaria* × *ananassa*) caused by the soilborne fungal pathogen *Fusarium oxysporum* f. sp. *fragariae*, has emerged over the past 20 years as a leading cause of severe plant wilt and death in California and many other parts of the world. We previously described several sources of resistance to race 1 of the pathogen; showed that resistance was conferred by dominant resistance genes (*R*‐genes) on chromosomes 2B (*FW1*, *FW2*, and *FW5*), 1A (*FW3*), and 6B (*FW4*); and identified a cultivar (Earliglow) that was hypothesized to be a source of novel *R*‐genes. Earliglow $S_{1}$ progeny segregated 15 resistant:1 susceptible ($\chi^{2}=0.03;p=0.87$), the Mendelian distribution expected when the phenotypes are caused by unlinked dominant duplicate epistasis. Here, we show that Earliglow carries a dominant *R*‐gene (*FW6*) in the *FW1* cluster on chromosome 2B and an incompletely dominant *R*‐gene (*FW7*) on chromosome 2A, where Fusarium wilt *R*‐genes have not been previously reported. The effect of *FW7* was masked by the epistatic effect of *FW6*; this was determined by self‐pollinating an $S_{1}$ individual predicted to be homozygous for the recessive (susceptible) *FW6* allele and heterozygous for *FW7* alleles, creating and whole‐genome sequencing Fusarium wilt resistant and susceptible $S_{2}$ bulks, and physically mapping the *FW7* locus by bulked segregant analysis. Lastly, we identified candidate genes for *FW7*, in addition to highly predictive *FW6*‐ and *FW7*‐associated SNPs for marker‐assisted selection of *FW6* and *FW7* alleles. This study laid the foundation for identifying the causal gene underlying *FW7* and increasing the durability of resistance to Fusarium wilt by pyramiding *FW7* with independent *R*‐genes.

## Core Ideas

- The resistance of the heirloom cultivar Earliglow to Fusarium wilt race 1 is conferred by a dominant gene on chromosome 2B (*FW6*) and a nearly additive gene on chromosome 2A (*FW7*).
- The effect of *FW7* was weaker than and masked by the epistatic effect of *FW6*.
- Whole‐genome sequencing bulked segregant analysis (WGS‐BSA) was validated as an approach for discovering and physically mapping DNA markers associated with large‐effect loci in octoploid strawberry.
- Highly predictive *FW6*‐ and *FW7*‐associated DNA markers were identified by genome‐wide association studies and WGS‐BSA.
- *FW6* and *FW7* can be pyramided to increase the durability of resistance to Fusarium wilt race 1.

## Plain Language Summary

Fusarium wilt, a strawberry disease that causes wilting and death in susceptible genotypes, has become an increasingly serious problem in home gardens and farms around the world. There are, however, natural genetic sources of resistance to the causal pathogen (*Fusarium oxysporum* f. sp. *fragariae*), including wild relatives and heirloom and modern cultivars. We previously identified genes on three chromosomes (1A, 2B, and 6A) that confer resistance to the most common race of the pathogen found in California (race 1). Using insights gained from previous studies, we discovered that the heirloom cultivar Earliglow carries a novel race 1 resistance gene on chromosome 2A (*FW7*). The effect of this newly identified resistance gene was hidden by the epistatic effect of a dominant gene (*FW6*) found in a previously identified cluster of resistance genes on chromosome 2B. We uncovered and physically mapped *FW7* using high‐throughput sequencing of resistant and susceptible bulks in combination with physical mapping of sequences to an octoploid reference genome, a tried‐and‐true approach known as bulked segregant analysis. Our study shows that strawberry has evolved multiple Fusarium wilt resistance genes that can be combined through marker‐assisted selection to prevent losses to the disease and increase the durability of resistance.

**Abbreviations**
BSA: bulked segregant analysis
*Fof*
: *Fusarium oxysporum*
f. sp.: *fragariae*
GBS: genotyping‐by‐sequencing
LD: linkage disequilibrium
QTL: quantitative trait locus

## 1 INTRODUCTION

*Fusarium oxysporum* Schltdl. is a diverse and widespread soilborne fungal pathogen that causes destructive vascular wilt diseases in susceptible genotypes of numerous economically important plants, including strawberry (*Fragaria* × *ananassa* Duchesne ex Rozier) (Edel‐Hermann & Lecomte, 2019; Gordon, 2017; Koike et al., 2009). The strawberry disease is specifically caused by different races of *Fusarium oxysporum* f. sp. *fragariae* (*Fof*) Winks & Williams (Dilla‐Ermita et al., 2023; Gordon, 2017; Henry et al., 2017, 2021; Winks & Williams, 1965). Fusarium wilt was first reported on strawberry in eastern Australia in the early 1960s and subsequently in East Asia (C. Kim et al., 1982; Koike et al., 2009; Koike & Gordon, 2015; Okamoto et al., 1970; Winks & Williams, 1965). The disease first surfaced on strawberry in California in 2005 and has since become one of the leading causes of plant wilt and death in California, primarily because susceptible cultivars are still widely grown even though resistant cultivars have been developed and are readily available (Cole, Knapp, et al., 2025; Cole, Pincot, et al., 2025; Koike & Gordon, 2015; Knapp et al., 2023, 2025). Their development was facilitated by the discovery and rapid deployment of *FW1*, a dominant gene that confers resistance to race 1 (Pincot et al., 2018, 2022), the *Fof* race most commonly found in California (Dilla‐Ermita et al., 2023; Henry et al., 2017).

*FW1* is one of three dominant, race‐specific resistance genes *(R‐genes)* identified and mapped to a near‐telomeric cluster on chromosome 2B (Pincot et al., 2022), including *FW2* and *FW5*. *FW2* was discovered in the heirloom cultivar Guardian, and *FW5* was discovered in a *Fragaria chiloensis* ecotype (PI602575). Whether *FW1*, *FW2*, and *FW5* are alleles or tandemly duplicated paralogs remains unknown. The *FW1* gene was discovered in modern cultivars developed at the University of California, Davis (Pincot et al., 2018), a factor that expedited the development and deployment of race 1 resistant cultivars through marker‐assisted selection (MAS) (Cole, Knapp, et al., 2025; Cole, Pincot, et al., in press; Knapp et al., 2023, 2025; Pincot et al., 2022). This was significant because most cultivars were found to be susceptible (*fw1/fw1*), and the frequency of the favorable allele (*FW1*) was only 0.16 in elite genetic resources developed at the University of California, Davis, before the discovery of *FW1* in 2016 (Pincot et al., 2018).

The abundant genetic diversity of strawberry has provided a strong foundation for developing Fusarium wilt resistant cultivars. Pincot et al. (2022) described over 200 sources of resistance to race 1, including *F. chiloensis* and *Fragaria virginiana* ecotypes and heirloom and modern cultivars. The heirloom cultivar Wiltguard was found to carry a dominant *R*‐gene on chromosome 1A (*FW3*), whereas the *F. virginiana* subsp. *virginiana* ecotype PI552277 was found to carry a dominant *R*‐gene on chromosome 6B (*FW4*). Hence, three independent Fusarium wilt resistance loci have previously been identified in strawberry, at least one of which could be a cluster of duplicated *R*‐genes on chromosome 2B.

The durability of the strawberry race 1 *R*‐genes that have either been widely deployed (*FW1*) or could be deployed (*FW2*–*FW5*) remains unclear and largely depends on the speed with which novel virulent races might evolve and defeat known *R*‐genes (Cowger & Brown, 2019; Henry et al., 2017; Mundt, 2014, 2018). Even though *FW1*, *FW2*, and *FW5* confer resistance to race 1, we sought to identify independent *R*‐genes in our previous studies that could be individually deployed or pyramided to increase the durability of resistance to race 1. *FW3* and *FW4* emerged from those studies (Pincot et al., 2022). Theoretically, pyramids multiplicatively decrease the probability of mutations independently evolving in the pathogen that simultaneously defeat every *R*‐gene in the pyramid (Mundt, 2018). To build on our earlier work and search for additional novel *R*‐genes, we undertook genetic studies of the cultivar Earliglow (Hokanson & Finn, 2000; Pincot et al., 2022). Earliglow was selected for study because Earliglow $S_{1}$ progeny were previously shown to segregate 15 resistant:1 susceptible (Pincot et al., 2022), the Mendelian distribution expected when the observed phenotypes are caused by unlinked dominant duplicate epistasis (Phillips, 1998). We concluded that Earliglow (the $S_{0}$ parent) must be heterozygous for two independent *R*‐genes and that they would either be independent or alleles or paralogs of *R*‐genes previously identified on chromosomes 1A, 2B, and 6B. In addition, Earliglow was one of only three individuals previously found to be resistant to *Fof* races from California, Australia, and Japan (Henry et al., 2021). This paper describes the California race 1 *R*‐genes found in Earliglow and sheds light on the nature of the epistatic interaction between them.

Genetic mapping in octoploid strawberry was revolutionized by the sequencing of the octoploid reference genome (Edger et al., 2019), thereby allowing for the use of sequence‐based genotyping approaches to simultaneously discover and physically map trait‐associated genetic variants while overcoming ascertainment biases and other limitations of genotyping arrays (Davey et al., 2011; Kockum et al., 2023). High‐throughput, short‐read DNA sequencing has become a standard approach for genotyping‐by‐sequencing (GBS) and identifying trait‐associated genetic variants in diploids and polyploids alike, with various applications such as genome‐wide association studies and bulked segregant analysis (BSA) (Davey et al., 2011; Han et al., 2015; Kockum et al., 2023; Liang et al., 2020; Uffelmann et al., 2021). The application of these approaches became feasible and straightforward in octoploid strawberry once the genome was initially sequenced (Edger et al., 2019). Importantly, Hardigan et al. (2020) showed that there was sufficient evolutionary divergence among the A, B, C, and D genomes to accurately assign 80%–85% of short‐read DNA sequences to physical addresses in an octoploid reference genome using standard diploid DNA sequence alignment tools (Glaubitz et al., 2014; H. Li & Durbin, 2009; H. Li et al., 2009). This paved the way for applying short‐read sequencing to a wide range of problems in octoploid strawberry studies where DNA or RNA sequences needed to be physically mapped, including GBS (Hardigan et al., 2020, Hardigan, Lorant, et al., 2021).

In this study, we validated the utility of whole‐genome sequencing bulked segregant analysis (WGS‐BSA) for identifying and physically mapping genetic variants in linkage disequilibrium (LD) with genes of interest in octoploid strawberry. We applied WGS‐BSA to the specific problem of uncovering and physically mapping a Fusarium wilt *R*‐gene (*FW7*) that was masked by duplicate dominant epistasis in an Earliglow $S_{1}$ family. We show that Earliglow (the $S_{0}$ parent) is heterozygous for a dominant *R*‐gene (*FW6*) in the *FW1* cluster on chromosome 2B, in addition to *FW7*, a newly identified incompletely dominant *R*‐gene on chromosome 2A. Lastly, we identify genetic variants that can be targeted by MAS to deploy and pyramid *FW7* with independent *R*‐genes as part of a strategy for increasing the durability of resistance to Fusarium wilt in strawberry.

**Core Ideas**

- The resistance of the heirloom cultivar Earliglow to Fusarium wilt race 1 is conferred by a dominant gene on chromosome 2B (*FW6*) and a nearly additive gene on chromosome 2A (*FW7*).
- The effect of *FW7* was weaker than and masked by the epistatic effect of *FW6*.
- Whole‐genome sequencing bulked segregant analysis (WGS‐BSA) was validated as an approach for discovering and physically mapping DNA markers associated with large‐effect loci in octoploid strawberry.
- Highly predictive *FW6*‐ and *FW7*‐associated DNA markers were identified by genome‐wide association studies and WGS‐BSA.
- *FW6* and *FW7* can be pyramided to increase the durability of resistance to Fusarium wilt race 1.

## 2 MATERIALS AND METHODS

### 2.1 Plant material

The plant materials for our study were developed using Earliglow, a *Fragaria* × *ananassa* short‐day cultivar (Scott & Draper, 1975). A clone of Earliglow (PI551394) was originally obtained from the USDA‐ARS National Clonal Germplasm Repository, Corvallis, OR (https://www.ars.usda.gov/pacific‐west‐area/corvallis‐or/national‐clonal‐germplasm‐repository/). Clones of the original Earliglow clone were annually propagated by rooting daughter plants in a field nursery at the University of California, Davis Wolfskill Experiment Orchard, Winters, CA.

We developed seed for an Earliglow $S_{1}$ family by manually self‐pollinating flowers on a single greenhouse‐grown Earliglow $S_{0}$ plant over the winter of 2017–2018. We developed seed for an Earliglow $S_{2}$ family by self‐pollinating flowers on a single greenhouse‐grown Earliglow $S_{1}$ plant over the winter of 2022–2023. The $S_{0}$ and $S_{1}$ flowers selected for self‐pollination were covered with butterfly cages to prevent cross‐contamination with pollen from other plants. The Earliglow $S_{0}$ parent plant was resistant to race 1 and predicted to be heterozygous for resistant and susceptible *FW6* and *FW7* alleles. The locus and allele names used here were not known a priori but were proposed once they were identified in our genetic analyses. The hypothesized genotype of the $S_{0}$ parent was *FW6/fw6 FW7/fw7*, where *FW6* and *FW7* are resistant (favorable) alleles and *fw6* and *fw7* are susceptible (unfavorable) alleles. The selected $S_{1}$ individual was resistant to race 1 and predicted to be homozygous for the susceptible *FW6* allele (*fw6/fw6*) and heterozygous for resistant and susceptible *FW7* alleles (*FW7/fw7*). Using *FW6*‐ and *FW7*‐associated single nucleotide polymorphisms (SNPs) genotyped with a 50K array (Hardigan et al., 2020), the predicted genotype of the $S_{1}$ parent was *fw6/fw6 FW7/fw7*.

$S_{1}$ and $S_{2}$ seeds were obtained by harvesting ripe fruit that were macerated in a pectinase solution (0.6 g/L) to separate achenes (seeds) from receptacles. Seeds were scarified by soaking in a 36 N sulfuric acid solution for 16 min, then were rinsed and immediately planted and germinated in peat pellets (Jiffy). $S_{1}$ seedlings were grown in a Winters, CA, greenhouse for 4 months before they were artificially inoculated with the pathogen before being transplanted to a Davis, CA, field in April 2022.

$S_{2}$ seedlings were grown indoors under fluorescent lighting 14 weeks before they were artificially inoculated with the pathogen, transplanted to four parts sphagnum peat moss:one part perlite (Sunshine Mix 1; Sun Gro Horticulture) in 10.2 cm × 10.2 cm × 15.2 cm plastic pots (T.O. Plastics), and grown in a growth chamber under a 12‐h daylength with a daytime temperature of 28°C and nighttime temperature of 20°C.

### 2.2 Artificial inoculation protocol

Plants were artificially inoculated with the AMP132 isolate of *Fof* using previously described protocols (Henry et al., 2021; Pincot et al., 2018). The AMP132 spores used for our work were obtained from Dr. Thomas R. Gordon at the University of California, Davis, Plant Pathology Department, and the origin of this isolate is described in Henry et al. (2017) and Henry et al. (2021). The pathogen was grown on potato dextrose agar under continuous fluorescent lighting at room temperature (approximately 22°C). Spores were released by scraping the plate surface in sterile water. Crude suspensions were then passed through two layers of sterilized cheesecloth to remove hyphae. Spore densities were estimated using a hemocytometer and diluted with sterile deionized water to a final density of 5 ×10^6^ spores/mL. The roots of each seedling were soaked in 40 mL of spore suspension for 7 min before planting.

### 2.3 Earliglow $S_{1}$ resistance screening experiment

The Earliglow $S_{1}$ family comprised 326 individuals that were genotyped and phenotyped for resistance to AMP132 (Supporting Information S1 and S2). Four‐month‐old seedlings were screened in a field experiment and arranged in an augmented randomized complete block design with four replications of two resistant check cultivars (Earliglow and Fronteras) and one susceptible check cultivar (Albion). Check cultivars were randomized within blocks using the R package *agricolae* (De Mendiburu & Simon, 2015).

Our Earliglow $S_{1}$ resistance screening experiment was conducted at the UC Davis Plant Pathology Farm. Strawberries had not been previously grown in the field selected for study. The fields were tilled and disced before fumigation and broadcast‐fumigated in October with a 60:40 mixture of chloropicrin:1,3‐dichloropropene (Pic‐Chlor 60, Cardinal Professional Products) at 560.4 kg/ha. The entire field was sealed with an impermeable plastic film for 1 week post‐fumigation before shaping 15.3 cm tall × 76.2 cm center‐to‐center raised beds. Subsurface irrigation drip tape was installed longitudinally along the beds followed by black plastic mulch with a single row of planting holes spaced 30.5 cm apart. Artificially inoculated plants were transplanted in April of 2022. The field was fertilized with approximately 198 kg/ha of nitrogen over the growing season and irrigated as needed to prevent water stress.

### 2.4 Earliglow $S_{2}$ resistance screening experiment

The Earliglow $S_{2}$ family comprised 90 individuals that were genotyped and phenotyped for resistance to AMP132 at the University of California, Davis, Controlled Environment Facility (Supporting Information S3–S5). Four‐month‐old seedlings were screened in a growth chamber experiment by artificial inoculation and were planted in July 2023 into 10.2 × 10.2 × 15.2 cm plastic pots filled with four parts sphagnum peat moss:one part perlite (Sunshine Mix 1; Sun Gro Horticulture). The seedlings plus two mock‐inoculated and two inoculated single‐plant replicates of Earliglow, a resistant control (Fronteras), and a susceptible control (Royal Royce) were arranged in an augmented randomized complete block design. The plants were grown under a 12‐h photoperiod with a 20°C night temperature and 28°C day temperature and irrigated with a dilute nutrient solution as needed to maintain adequate soil moisture.

### 2.5 Disease resistance phenotyping

The individuals in these studies were visually phenotyped for resistance to Fusarium wilt over multiple postinoculation time points using an ordinal disease rating scale from 1 to 5, where 1 = *no symptoms*; 2 = *stunting, deformation, some chlorosis*; 3 = *chlorosis, wilt, some leaf dieback*; 4 = *heavy stunting, severe wilt, most leaves dead*; and 5 = *dead* (Gordon, 2017; Henry et al., 2017, 2021; Pincot et al., 2018). Both field and growth chamber studies were phenotyped weekly for 6–12 weeks postinoculation, and the onset and progression of disease symptoms among resistant and susceptible checks were used as guides for initiating and terminating phenotyping.

### 2.6 SNP genotyping and sequencing

DNA was isolated from newly emerging leaves using a previously described protocol (Pincot et al., 2018). Leaf samples were placed into coin envelopes and freeze‐dried in a Benchtop Pro lyophilizer (VirTis SP Scientific). Approximately 0.2 g of dried leaf tissue per sample was placed into wells of 2.0 mL 96‐well deep‐well plates. Tissue samples were ground using stainless steel beads in a Genogrinder Mini 1600 (SPEX Sample Prep). Genomic DNA was extracted from powdered leaf samples using the E‐Z 96 Plant DNA Kit (Omega Bio‐Tek) according to the manufacturer's instructions. To enhance the DNA quality and yield and reduce polysaccharide carry‐through, the protocol was modified by adding Proteinase K to the lysis buffer to a final concentration of 0.2 mg/mL and extending lysis incubation to 45 min at 65°C. Once the lysate separated from the cellular debris, RNA was removed by adding RNase A. The mixture was incubated at room temperature for 5 min before a final spin down. To ensure high DNA yields, the sample was incubated at 65°C for 5 min following the addition of elution buffer. DNA quantification was performed using Quantiflor dye (Promega) on a Synergy HTX (Biotek).

The Earliglow $S_{1}$ and $S_{2}$ families were genotyped with a 50K Axiom SNP array (Hardigan et al., 2020) (Supporting Information S2 and S4). The probe DNA sequences for SNP markers on the 50K Axiom array were previously anchored to the Camarosa and Royal Royce reference genomes (Edger et al., 2019; Hardigan et al., 2020, Hardigan, Feldmann, et al., 2021). The physical addresses for the SNP markers were determined by Pincot et al. (2022). The results in this paper utilized the haplotype‐resolved Royal Royce reference genome FaRR1 (Hardigan, Lorant, et al., 2021) unless otherwise noted. SNP genotypes were called using the Affymetrix Axiom Suite (v1.1.1.66). Samples with call rates exceeding 89%–93% were included in genetic analyses.

The Earliglow $S_{2}$ family was divided into resistant and susceptible bulks based on the final time point (FTP) disease score. The resistant bulk consisted of 29 individuals with an FTP disease score of 1, and the susceptible bulk consisted of 22 individuals with an FTP disease score of 5. Individuals with a FTP disease score of 2, 3, or 4, and 26 individuals that reached a disease score of 5 earlier than expected (by 5 weeks post‐inoculation) were not included in the bulks. The resistant and susceptible bulks were constructed by mixing equivalent amounts of DNA from each of the 29 resistant and 22 susceptible individuals. Paired‐end sequencing libraries were generated for the bulks using Illumina NovaSeq X Plus (Illumina Inc). Raw reads were submitted to the NCBI SRA database under BioProject number PRJNA1251051.

### 2.7 Statistical analyses

Pairwise LD measurements for 76 of the 327 SNPs on chromosome 2B from the Earliglow $S_{1}$ family were computed and visualized with the R package *LDheatmap* (https://rdrr.io/cran/LDheatmap/; Shin et al. 2006). *FW6*‐ and *FW7*‐associated SNPs with the highest ‐log 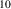 (FDR *p*‐values) in the respective peaks were selected to partition the associated locus effect on disease score. The disease scores observed in the Earliglow $S_{1}$ and $S_{2}$ families were analyzed using linear mixed models in the R package *lme4* (https://cran.r‐671project.org/web/packages/lme4/index.html) (Bates et al., 2015). The raw phenotypic data from both families were analyzed and modeled as follows: y=FW6+FW7+FW6:FW7, where y is the phenotypic observations, one locus was fixed and the other was a random effect, and FW6:FW7 was a random effect. Estimated marginal means (EMMs) were estimated using the R package *emmeans* (Lenth, 2024). Variance components for random effects were estimated using restricted maximum likelihood (REML) (Cheung, 2013). The phenotypic variance explained by each marker was assessed (PVE = ${\hat{\sigma }}_{M}^{2}/{\hat{\sigma }}_{P}^{2})$, where ${\hat{\sigma }}_{M}^{2}$ is the bias‐corrected average semivariance REML estimate of the marker genetic variance and ${\hat{\sigma }}_{P}^{2}$ refers to the phenotypic variance (Feldmann et al., 2021). The additive effect ($\hat{a}$) was estimated by $\hat{a}$ = (${\bar{y}}_{++}$ ‐ ${\bar{y}}_{--}$)/2, the dominance effect ($\hat{d}$) was estimated by $\hat{d}$ = ${\bar{y}}_{+-}$ ‐ (${\bar{y}}_{++}$ + ${\bar{y}}_{--}$)/2, and the degree of dominance was estimated by $|\hat{d}/\hat{a}|$ where ${\bar{y}}_{++}$, ${\bar{y}}_{+-}$, and ${\bar{y}}_{--}$ are the EMMs for individuals with resistant (+) or susceptible (−) alleles at each locus (Falconer, 1996; Walsh, 2001).

### 2.8 Genetic mapping

SNP markers with ≤ 5% missing data, high‐quality codominant genotypic clusters, progeny genotypes concordant with parent genotypes, and non‐distorted segregation ratios (*p* > 0.01) were utilized for genetic and quantitative trait locus (QTL) mapping analyses. SNP markers were genetically mapped in the $S_{1}$ and $S_{2}$ families using phase‐known backcross mapping functions from the subset of SNPs that were heterozygous in the respective resistant parents. Genetic maps were constructed using the R packages *onemap* and *BatchMap* (Margarido et al., 2007; Schiffthaler et al., 2017) as described in Pincot et al. (2022).

### 2.9 QTL mapping

The effects of individual SNP marker loci were estimated using single marker regression as implemented in the R package *qtl* (Broman et al., 2003). The test statistics were plotted against physical positions in the Royal Royce reference genome that were corrected using genetic mapping information. For the Earliglow $S_{2}$ family, the physical positions of markers on linkage groups 2A_I and 2A_II were determined by BLAST against the Earliglow genome assembly. Genome‐wide significance thresholds (*p* = 0.05) were calculated by permutation testing with 2000 permutations (Sen & Churchill, 2001). We estimated 1‐LOD (likelihood odd) support intervals and 95% Bayesian credible intervals for the *FW6* QTL using the *bayes.int* function (Broman & Sen, 2009).

### 2.10 Kompetitive allele specific primer marker development

Kompetitive allele specific primer (KASP) markers were developed for three Axiom 50K array SNPs strongly associated with the *FW6* locus on chromosome 2B (Semagn et al., 2014). The array IDs for the SNPs we targeted were AX‐184258944 (G/A; 670,184), AX‐184415132 (C/T; 698,410), and AX‐184945734 (C/T; 752,279). KASP primers were designed using PolyOligo with physical positions in the Royal Royce reference genome (Ledda et al., 2019) (https://github.com/MirkoLedda/polyoligo). We used default PolyOligo design parameters and only tested primers for KASP markers with a heuristic quality score of 10 (*highest quality*) on a scale from 1 (*lowest quality*) to 10. The allele‐specific forward and common reverse primer sequences for these KASP markers are shown in Supporting Information S6. These KASP markers were initially tested and validated by screening 22 Earliglow $S_{1}$ individuals that were predicted to be homozygous for resistant or susceptible *FW6* alleles using the three array SNPs. Earliglow $S_{2}$ individuals were genotyped with the three KASP markers to confirm that they were homozygous for the susceptible *FW6* allele (Supporting Information S6). KASP assays were performed as described by LGC Biosearch Technologies (https://biosearchtech.com/support/education/kasp‐genotyping‐reagents/kasp‐overview). To ensure DNA stability, 1uL of 8% polyvinylpyrrolidone was added to each reaction with 4uL of DNA and 0.14uL KASP assay mix (KBS‐1050‐121). KASP genotyping was performed on a ThermoFisher QuantStudio3, and further polymerase chain reaction cycling was performed to increase amplification, when needed.

### 2.11 Earliglow genome assembly and annotation

Newly emerging leaf tissue was collected from Earliglow and immediately flash‐frozen in liquid nitrogen for high‐molecular weight DNA extraction. DNA extraction and PacBio HiFi sequencing was performed by Arizona Genomics Institute (Tuscon, AZ) at 20x depth. Earliglow HiFi reads were assembled with Hifiasm (v0.19.9‐r616) (Cheng et al., 2021, 2022). The contigs were then scaffolded with the Royal Royce genome as reference with RagTag “scaffold” (Alonge et al., 2022). A Benchmarking Universal Single‐Copy Orthologs (BUSCO) analysis was performed using default parameters to assess the “core eudicot” gene set (Supporting Information S7). Both Earliglow haplotypes (Earliglow1 and Earliglow2) were submitted to NCBI Genome Database under BioProject number PRJNA1253726.

For annotation, the Earliglow genome was first masked using RepeatMasker (http://www.repeatmasker.org) and a custom library of strawberry genomic repeats (Hardigan, Feldmann, et al., 2021). We then annotated using the Docker container of BRAKER3 (Bruna et al., 2024; Buchfink et al., 2015; Gabriel et al., 2021, 2024; Kim et al., 2019; H. Li et al., 2009; Leinonen et al., 2011; Pertea & Pertea, 2020; Quinlan, 2014; Stanke et al., 2008, 2006). Briefly, an Augustus model for strawberry was developed using a diverse set of published RNA‐seq data, including leaves, roots, and runners, under unstressed and abiotic/biotic stressed conditions (SRR7157738, SRR7157740, ERR1817363, ERR1817364, ERR1817365, ERR1817366, ERR1817367, ERR1817368, SRR8298768, SRR8298767, SRR8298772, SRR8298771, and SRR8298763) (Edger et al., 2019; Z. Li et al., 2019; Sánchez‐Sevilla et al., 2017). BRAKER3 was then run with these same RNA‐seq datasets as transcriptome hints, the orthoDB complete protein database as protein hints, and the pretrained Augustus model, yielding the predicted annotations used in this paper.

### 2.12 Bulked segregant analysis

Sequencing libraries from resistant and susceptible Earliglow $S_{2}$ bulks were aligned to the Earliglow genome using the Burrows‐Wheeler Aligner software. Duplicates were marked by picard (v2.26.2) and alignments were filtered for quality scores > 40. Variants were called for each bulk with freebayes (v1.3.6) (Garrison & Marth, 2012) and filtered to only include variants where the alternate allele was observed in the resistant bulk. Variants were subsequently filtered with QTLseqr (minTotalDepth = 50, maxTotalDepth = 300, depthDifference = 100, minSampleDepth = 20) (Mansfeld & Grumet, 2018) and QTL were identified using the G prime method (windowSize = 1e6, outlierFilter = deltaSNP) (Magwene et al., 2011) (Supporting Information S5).

### 2.13 *FW6* haplotype analysis

The Earliglow pedigree was determined from Pincot et al. (2021) and Scott and Draper (1975). Genotypic data were phased using Beagle 5.4v (Browning et al. 2021) with the software's default parameters and minor allele frequency of 0.3. Hierarchical clustering was performed on genotypic data from the first 2 Mb on chromosome 2B using the R function *hclust* with the “average” method.

### 2.14 Candidate *FW7* gene identification

We utilized the functional annotations of the Royal Royce reference genome to identify candidate *FW7* genes. Orthologs in the Royal Royce genome were identified using GENESPACE (Lovell et al., 2022). Candidate *FW7* genes were identified by identifying genes in the *FW7* region functionally annotated as encoding nucleotide‐binding leucine‐rich repeat (NLR) proteins or pattern‐recognition receptors. Protein domain structure validation of candidate genes was performed with InterPro (Paysan‐Lafosse et al., 2023). Candidate genes for *FW6* (predicted in the present study to be *FW2*) were described by Pincot et al. (2022) and are under separate investigation; hence, we are only reporting candidate genes here for the newly identified locus (*FW7*).

## 3 RESULTS

### 3.1 An Earliglow $S_{1}$ family segregated for independent Fusarium wilt *R*‐genes

**FIGURE 1:**
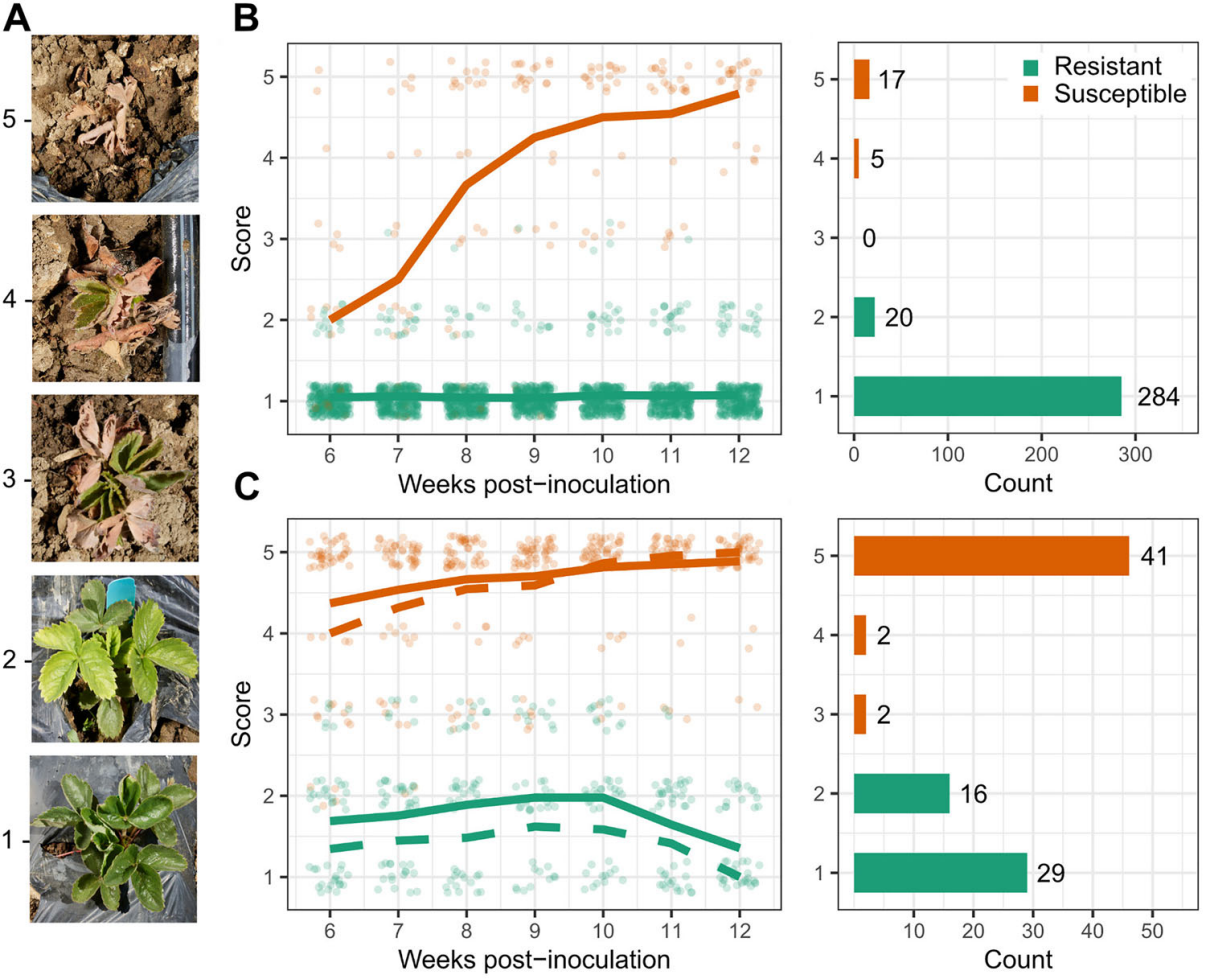
Fusarium wilt race 1 resistance phenotypes observed among Earliglow $S_{1}$ and $S_{2}$ progeny. (A) The range of disease symptoms observed at 10 weeks post‐inoculation among Earliglow $S_{1}$ progeny and associated ordinal disease scores, where 1 = *symptomless* and 5 = *dead*. $S_{1}$ and $S_{2}$ individuals were inoculated with the AMP132 isolate of *Fusarium oxysporum* f. sp. *fragariae*. (B) The distribution of ordinal disease scores observed among 326 $S_{1}$ individuals over 6–12 weeks postinoculation (left) and counts of $S_{1}$ individuals classified as resistant or susceptible at 12 weeks postinoculation (right). (C) The distribution of ordinal disease scores observed among 90 $S_{2}$ individuals over 6–12 weeks post‐inoculation (left) and counts of $S_{2}$ individuals classified as resistant or susceptible at 12 weeks post‐inoculation (right).

We phenotyped 326 4‐month‐old seed‐propagated Earliglow $S_{1}$ individuals for Fusarium wilt race 1 disease symptoms observed 6–12 weeks postinoculation (Figure 1A,B; Supporting Information S1). Symptoms were observed on plants artificially inoculated with AMP132, a California race 1 isolate of the pathogen (Henry et al., 2021). The progression of disease symptoms and distribution of resistant and susceptible $S_{1}$ individuals at 12 weeks postinoculation suggested that two dominant *R*‐genes segregated in the Earliglow $S_{1}$ family (Figure 1B). The resistant and susceptible phenotypic classes were distinct (nonoverlapping) at 12 weeks postinoculation. The disease symptom ratings (y) of $S_{1}$ individuals classified as resistant were constant over time with 284 individuals exhibiting no symptoms (y=1) and 20 displaying mild symptoms (y=2) (Figure 1A,B). Conversely, the disease symptoms of susceptible $S_{1}$ individuals worsened over time with five nearly killed (y=4) and 17 killed (y=5) by 12 weeks post‐inoculation (Figure 1A,B). Using these categorical classifications, the observed segregation ratio in the $S_{1}$ family (304 resistant:22 susceptible) was not significantly different from 15 resistant:1 susceptible ($\chi^{2}=0.03;p=0.87$), the Mendelian distribution expected when the observed phenotypes are caused by dominant duplicate epistasis (Phillips, 1998).

### 3.2 Earliglow carries a dominant *R*‐gene (*FW6*) that physically mapped to a previously identified cluster of dominant *R*‐genes on chromosome 2B

**FIGURE 2:**
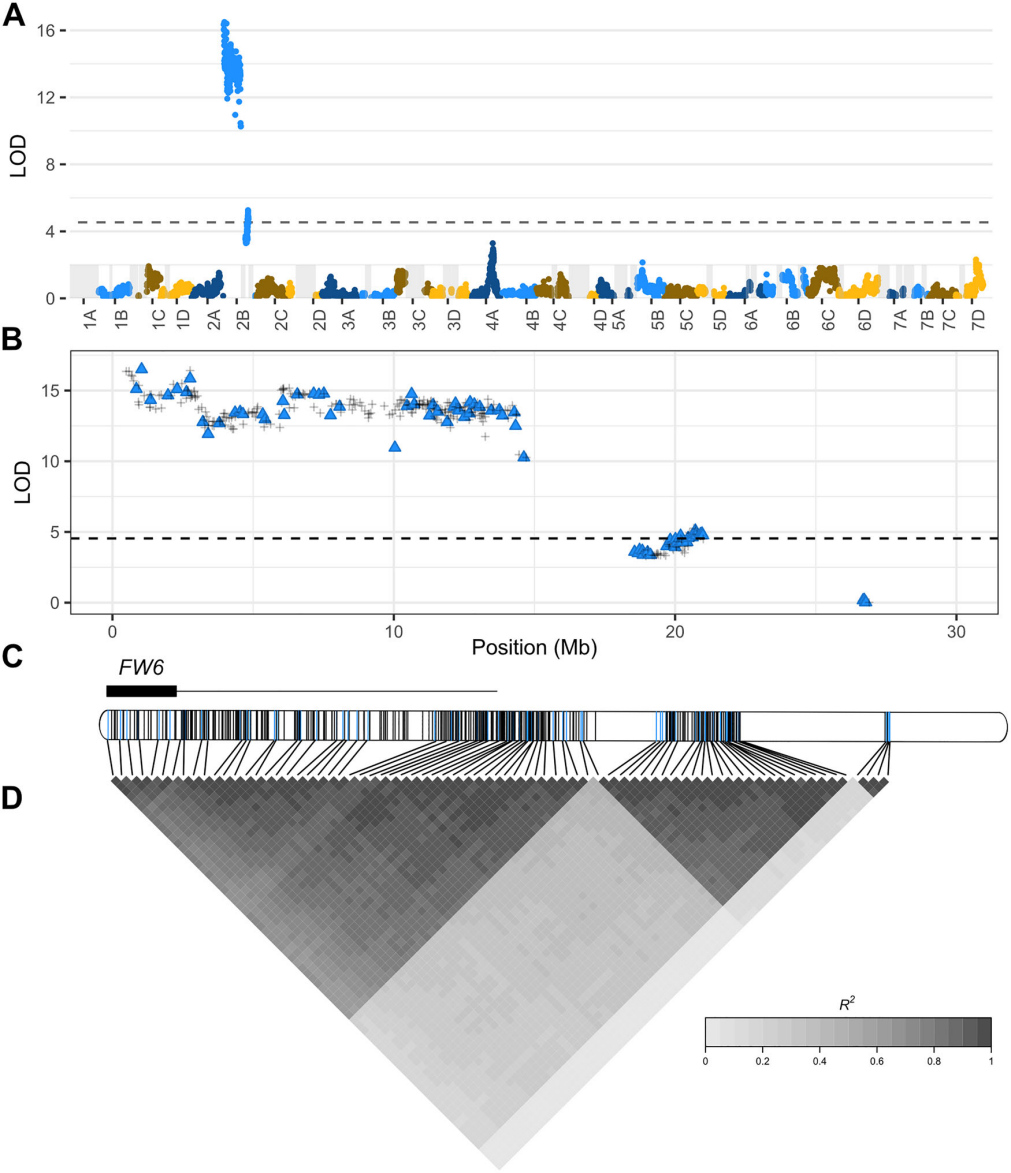
Genome‐wide search for associations between 50K array‐genotyped single nucleotide polymorphisms (SNPs) and genes conferring resistance to Fusarium wilt race 1. (A) Likelihood odds (LODs) for single‐marker analyses of associations between SNP marker loci and Fusarium wilt race 1 resistance phenotypes in the Earliglow $S_{1}$ family (n=331). LODs are plotted against physical positions of SNP marker loci across the Royal Royce genome. The physical positions and chromosome assignments of SNPs used in the analysis were corroborated by de novo genetic mapping. The horizontal dashed line demarcates a genome‐wide LOD significance threshold of 4.54. The vertical gray bars identify runs‐of‐homozygosity spanning a minimum of 35 physically anchored SNPs. (B) LODs plotted against physical positions of 381 SNP marker loci on chromosome 2B in Royal Royce genome (depicted by crosses and triangles). Triangles indicate a subset of 76 SNPs selected for estimating pairwise linkage disequilibrium (LD) statistic ($R^{2}$). (C) SNP positions on chromosome 2B (blue color indicates 76 SNPs used in pairwise LD estimates), a 1‐LOD support interval for the *FW6* locus (box spanning 0.48–2.7 Mb), and a 95% Bayesian credible interval for the *FW6* locus (line spanning 0.48–13.3 Mb). (D) Pairwise estimates of the $R^{2}$ LD statistic among 76 SNPs on chromosome 2B.

The Earliglow $S_{1}$ family was genotyped with a 50K SNP array to identify SNPs in LD with the two *R*‐genes predicted by our Mendelian genetic analysis (Supporting Information S2). QTL mapping identified a single LD block of resistance score‐associated SNPs on chromosome 2B (Figure 2). The LOD statistics displayed in the Manhattan plot (Figure 2A) were estimated by single marker QTL analysis using the physical positions of SNPs in the Royal Royce genome (FaRR1; https://phytozome‐next.jgi.doe.gov/info/FxananassaRoyalRoyce_v1_0). As a consequence of limited recombination, significant associations were observed across the entire upper half of chromosome 2B (0.0–14.2 Mb; 47.6 cM) (Figure 2B). Long runs‐of‐homozygosity were observed downstream of the upper LD block (Figure 2B). Apart from two short LD blocks downstream (18.6–21.0 Mb and 25.1–27.2 Mb), the lower half of chromosome 2B was homozygous for 50K array‐genotyped SNPs with recombination coldspots separating the three LD blocks (Figure 2B–D).

The SNPs most strongly associated with resistance phenotypes mapped close to the upper telomere proximal to a cluster of three previously physically mapped *R*‐genes on chromosome 2B: *FW1*, *FW2*, and *FW5* (Pincot et al., 2018, 2022). This Earliglow *R*‐gene is hereafter designated *FW6*. The single‐most significant *FW6*‐associated SNP was AX‐184674612 (0.83 Mb; $\log_{10}(p-$ value) = 1.44 $\times 10^{-18}$). The pattern of LD decay suggested that *FW6* is a member of the *FW1* family of dominant *R*‐genes found on chromosome 2B (Figures 1 and 2). The allelic relationships among those *R*‐genes are not known. They could be alleles at a single locus, paralogs of closely linked, tandemly duplicated loci, or a combination thereof. As described below, our analyses of pedigree records and SNP haplotypes suggest that *FW6* is identical‐by‐descent to *FW2*, an *R*‐gene discovered in the cultivar Guardian (Pincot et al., 2022).

Our initial genome‐wide single‐marker QTL analysis did not uncover the physical location of the second *R*‐gene predicted to have segregated in the Earliglow $S_{1}$ population (hereafter designated *FW7*). To explore this further, we repeated the QTL analysis among 63 individuals in the $S_{1}$ family that were predicted to be homozygous for the recessive (susceptible) *FW6* allele; however, this did not identify SNPs associated with *FW7* either. We suspected that the effect of *FW7* was masked by the epistatic effect of *FW6* and that our $S_{1}$ family analysis was too underpowered to uncover the effect of *FW7* despite the 15 resistant:1 susceptible distribution suggesting that *FW7* was an independent dominant *R*‐gene (Figure 4).

**FIGURE 3:**
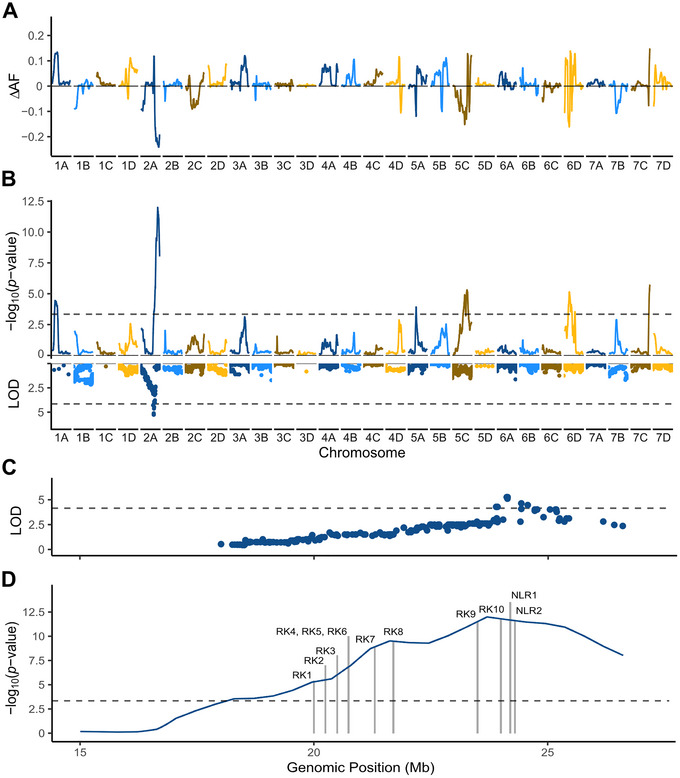
Quantitative trait locus (QTL) mapping and bulked segregant analysis (BSA) statistics for the Earliglow $S_{2}$ family. (A) Genome‐wide differences in single nucleotide polymorphism (SNP) allele frequencies (Δ AF) between resistant and susceptible $S_{2}$ bulks. (B) The *y*‐axis in the upper Manhattan plot displays the statistical significance of SNP allele frequency differences between resistant and susceptible $S_{2}$ bulks estimated by $G^{\prime }$ analysis. The horizontal dashed line displays a false discovery rate (FDR)‐corrected $-\log_{10}\left(\text{p-value}\right)$ threshold of 0.01. The *y*‐axis in the lower Manhattan plot displays the likelihood odd (LOD) scores for single marker QTL analyses of Fusarium wilt race 1 phenotypes in the Earliglow $S_{2}$ family. LODs are plotted against physical positions of 50K array‐genotyped SNPs. The horizontal dashed line displays the genome‐wide LOD significance threshold of 4.15. (C) LOD scores for single marker QTL analyses of Fusarium wilt race 1 phenotypes on chromosome 2A in the Earliglow $S_{2}$ family. (D) *G′* statistics for BSA‐identified SNPs on chromosome 2A. Genes with putative disease resistance functions are labeled (see Table 5 for annotation details).

**FIGURE 4:**
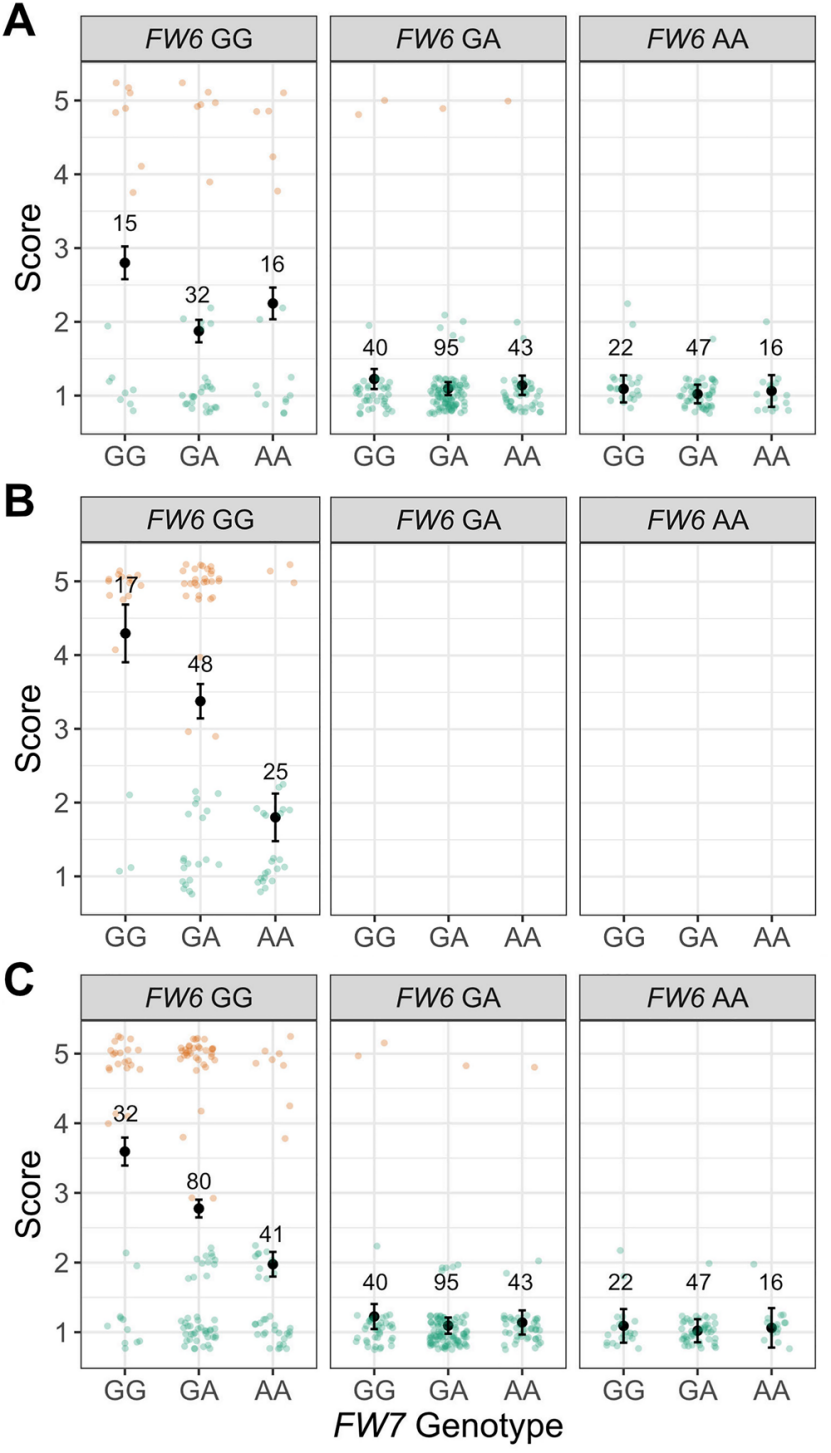
Fusarium wilt race 1 resistance score means for *FW6*‐ and *FW7*‐associated single nucleotide polymorphism (SNP) genotypes among Earliglow $S_{1}$ and $S_{2}$ progeny. The A/G SNP marker associated with *FW6* was AX‐184674612. The A/G SNP marker associated with *FW7* was AX‐184026067. The *A* alleles for both were associated with favorable *FW6* and *FW7* alleles. (A) Observed phenotypes and SNP genotype means for 326 $S_{1}$ progeny. (B) Observed phenotypes and SNP genotype means for 90 $S_{2}$ progeny. (C) Observed phenotypes and SNP genotype means for 326 $S_{1}$ and 90 $S_{2}$ progeny combined. Individuals classified as resistant are shown in green, whereas individuals classified as susceptible are shown in orange. The resistance score means and standard error bars are plotted for each of the nine AX‐184674612 × AX‐184026067 (*FW6* × *FW7*). Counts of individuals within each SNP genotypic class are shown above the means.

### 3.3 Whole‐genome sequencing BSA and QTL mapping in an Earliglow $S_{2}$ family uncovered *FW7*, an incompletely dominant *R*‐gene on chromosome 2A

To eliminate the confounding epistatic effect of the *FW6* allele and increase statistical power for mapping *FW7*, we created and analyzed an $S_{2}$ family (n=90 individuals) segregating for only *FW7* (Figure 1C; Supporting Information S3). This was done by identifying and self‐pollinating a resistant Earliglow $S_{1}$ individual predicted to be homozygous for the recessive (susceptible) *FW6* allele (*fw6/fw6*). The *FW6* genotypes of these individuals were predicted using 50K array‐genotyped SNPs associated with *FW6*, including the lead SNP (AX‐184674612) and six others in the upper LD block on chromosome 2B: AX‐184513681 (0.49 Mb), AX‐184522115 (0.59 Mb), AX‐184258944 (0.67 Mb), AX‐184415132 (0.69 Mb), AX‐184945734 (0.75 Mb), and AX‐184727873 (2.8 Mb) (Figures 1 and 2B). The $S_{2}$ family (n=90 individuals) was genotyped with KASP markers designed for three of these array SNPs to confirm the prediction that they were homozygous for the susceptible *FW6* allele (Supporting Information S6). The $S_{2}$ family, Earliglow, and resistant and susceptible check cultivars (Fronteras and Royal Royce, respectively) were artificially inoculated with the AMP132 isolate and phenotyped for resistance to race 1 over 6–12 weeks post‐inoculation in a growth chamber (Figure 1C; Supporting Information S3). Twenty‐six of the 90 $S_{2}$ progeny were eliminated from our analysis because they died substantially faster than normal (within 3–5 weeks post‐inoculation) and much faster than the susceptible check (10–12 weeks post‐inoculation) from causes that appeared to be unrelated to Fusarium wilt. Several of these individuals lacked vigor and could have been affected by inbreeding depression.

We observed a bimodal distribution of resistant and susceptible individuals in the $S_{2}$ family at 12 weeks post‐inoculation (Figure 1C). Earliglow and the resistant checks were symptomless, whereas the susceptible check was dead by the FTP (12 weeks post‐inoculation). The observed distribution in the $S_{2}$ family at the FTP (45 resistant:45 susceptible) was inconsistent with the segregation of a single dominant gene ($\chi^{2}=11.5;p=0.0007$). The pattern of disease symptom progression in the $S_{2}$ family was noisier than that observed in the $S_{1}$ family. Despite the absence of the expected 3 resistant:1 susceptible Mendelian phenotypic distribution, highly resistant (y=1) and highly susceptible (y=5) $S_{2}$ individuals were confidently identified, and the bimodal phenotypic distribution suggested that a large‐effect gene was segregating (Figure 1C). We investigated this with BSA and QTL mapping in the $S_{2}$ family.

To identify SNPs in LD with *FW7* and pinpoint the location of *FW7* in the octoploid genome, we assembled and whole‐genome sequenced DNA bulks of resistant (R) and susceptible (S) $S_{2}$ individuals for BSA (Michelmore et al., 1991). DNA was isolated from healthy leaf tissue before the plants were inoculated with the pathogen. We bulked DNA samples from 22 $S_{2}$ individuals that were killed by the disease and 29 $S_{2}$ individuals that were symptomless. Genetic variants were called between R‐ and S‐bulks by aligning DNA sequences to a phased‐scaffold Earliglow genome assembly (Figure 3; Supporting Information S5). After filtering, 2,484,661 genetic variants were called for genome‐wide analyses of allele frequency differences between R‐ and S‐bulks using $G^{\prime }$ statistics (Figure 3A; Figures S1 and S2). LD blocks of genetic variants with statistically significant allele frequency differences were identified on chromosomes 1A, 2A, 5A, 5B, 6D, and 7C; however, the strongest signals (largest allele frequency differences) were observed for SNPs on chromosome 2A (Figure 3B,D). We obtained identical results when DNA sequences for R‐ and S‐bulks were aligned to the haplotype‐phased Royal Royce genome. We identified 22,447 statistically significant genetic variants in the chromosome 2A LD block (Figure 3B,D). SNPs with the most significant allele frequency differences spanned 23.6–25.1 Mb on chromosome 2A (Figure 3B,D; Table 1). Thus, *FW7* physically mapped to chromosome 2A in our BSA.

**TABLE 1:** Single nucleotide polymorphisms (SNPs) with the greatest allele frequency differences between Fusarium wilt race 1 resistant and susceptible bulks of Earliglow $S_{2}$ progeny.

SNP^a^	Position (bp)^b^	Pr > $G^{\prime }$ c	$f_{R}$ (%)^d^	$f_{S}$ (%)^e^
C/A	23,682,324	2.29 $\times 10^{-12}$	88.9	87.5
C/A	23,722,633	2.08 $\times 10^{-12}$	96.2	65.2
T/A	25,076,436	1.57 $\times 10^{-11}$	80.0	95.5

^a^ For each SNP genotype, the first allele was the common allele in the resistant bulk, whereas the second allele was the common allele in the susceptible bulk.

^b^ The physical position of the *FW7*‐associated SNP in the Earliglow genome.

^c^ The $G^{\prime }$‐estimated probability that the allele frequency difference between resistant and susceptible bulks was random.

^d^ $f_{R}$ (%) is the frequency of the common allele in the resistant bulk.

^e^ $f_{S}$ (%) is the frequency of the common allele in the susceptible bulk.

To confirm the physical location of *FW7*, we applied QTL analysis to the $S_{2}$ family using 50K array SNP genotypes. This analysis identified a single LD block of resistance score‐associated SNPs on chromosome 2A in the $S_{2}$ family (Figure 3B). Significant associations were not observed anywhere else in the genome. The LOD statistics displayed in the Manhattan plot (lower panel of Figure 3B) were estimated by single‐marker QTL analysis using the physical positions of SNPs in the Royal Royce genome (Supporting Information S4). The 50K array SNPs most strongly associated with *FW7* spanned 23.9–24.4 Mb (Figure 3C). The most significant *FW7*‐associated SNP was AX‐184026067 (A/G; 24.1 Mb; $-\log_{10}(p-$ value) = 5.26). The *A* allele was associated with the favorable *FW7* allele, whereas the *G* allele was associated with the unfavorable *FW7* allele. The pattern of $G^{\prime }$‐statistic decay on chromosome 2A was identical to the pattern of LD decay observed in the QTL analysis of the $S_{2}$ family (Figure 3B,D). Thus, we confirmed that *FW7*, the *R*‐gene masked by the epistatic effect of *FW6* in the Earliglow $S_{1}$ family, is located on chromosome 2A (Figure 3B,C) and that Earliglow is the source of a novel Fusarium wilt *R*‐gene. Fusarium wilt race 1 *R*‐genes have not been previously identified on this chromosome (Pincot et al., 2022).

### 3.4 The effect of the favorable *FW7* allele is nearly additive in genetic backgrounds lacking other *R*‐genes

Once the physical location of the *FW7* locus was determined and *FW7*‐associated SNPs were identified, we revisited our original analysis and estimated the effects of *FW6*‐ and *FW7*‐associated SNPs on resistance phenotypes in the $S_{1}$ family. The *FW6*‐ and *FW7*‐associated SNPs selected as factors for one‐ and two‐locus analyses were AX‐184674612 and AX‐184026067, respectively (Tables 2–4; Figure 4). Our analyses showed that the effect of the favorable *FW6* allele was nearly completely dominant ($|\hat{d}/\hat{a}|=0.84$) and masked the effect of the favorable *FW7* allele. The phenotypic means for *FW6/FW6 FW7/FW7* ($\bar{y}=1.06$), *FW6/FW6 FW7/fw7* ($\bar{y}=1.02$), and *FW6/FW6 fw7/fw7* ($\bar{y}=1.09$) genotypes were not significantly different in the $S_{1}$ family. Although the *FW6* × *FW7* interaction effect was marginally nonsignificant in the $S_{1}$ family analysis (Pr > *F* = 0.11), the pattern observed among the two‐locus genotypic means substantiated the strength and nature of the epistatic interaction between *FW6* and *FW7* (Table 2; Figure 4).

**TABLE 2:** *F*‐statistics for linear mixed model (LMM) analyses of Fusarium wilt race 1 resistance score variation caused by the segregation of *FW6* and *FW7* among Earliglow $S_{1}$ and $S_{2}$ progeny.

Family^a^	n	Factor^b^	*F*	Pr > *F* c
Earliglow $S_{1}$	326	*FW6*	21.6	<0.0001
		*FW7*	6.0	0.0029
		*FW6* × *FW7*	1.9	0.11
Earliglow $S_{2}$	90	*FW7*	120.2	<0.0001
Earliglow $S_{1}$ and $S_{2}$	416	*FW6*	47.9	<0.0001
		*FW7*	18.4	<0.0001
		*FW6* × *FW7*	5.3	0.0003

^a^ The Earliglow $S_{1}$ family was developed by self‐pollinating the cultivar Earliglow, an ($S_{0}$) individual heterozygous for *FW6* and *FW7*. The Earliglow $S_{2}$ family was developed by self‐pollinating a single Earliglow $S_{1}$ individual that was homozygous for *FW6* and heterozygous for *FW7*.

^b^ The factors in linear mixed model analyses were Axiom 50K array SNP markers associated with the segregation of *FW6* (AX‐184674612) and *FW7* (AX‐184026067) in Earliglow $S_{1}$ and $S_{2}$ families. AX‐184674612 mapped to chromosome 2B, whereas AX‐184026067 mapped to chromosome 2A. *F*‐statistics are shown for LMM analyses of the effects of two interacting loci (*FW6* and *FW7*) among 326 $S_{1}$ progeny, the effect of a single locus (*FW7*) among 90 $S_{2}$ progeny, the effects of two interacting loci (*FW6* and *FW7*) among 326 $S_{1}$ and 90 $S_{2}$ progeny combined. The A/G SNP marker associated with the *FW6* locus was AX‐184674612. The A/G SNP marker associated with the *FW7* locus was AX‐184026067. The *A* alleles for both were associated with favorable *FW6* and *FW7* alleles.

^c^ Pr > *F* is the probability of observing a value of *F* as large as or larger than the one estimated under the null hypothesis of no effect of a locus (*FW6* or *FW7*) or no effect of the interaction between loci (*FW6* × *FW7*).

**TABLE 3:** Additive and dominance effects of *FW6*‐ and *FW7*‐associated single nucleotide polymorphisms (SNPs) among Earliglow $S_{1}$ and $S_{2}$ progeny.

			EMMs^c^			Contrasts^d^				
Family^a^	n	Locus^b^	${\bar{y}}_{++}$	${\bar{y}}_{+-}$	${\bar{y}}_{--}$	$\hat{a}$	Pr > *F*	$\hat{d}$	Pr > *F*	$|\hat{d}/\hat{a}|$
Earliglow $S_{1}$	326	*FW6*	1.05	1.13	2.19	−0.57	< 0.0001	0.48	< 0.0001	0.84
		*FW7*	1.36	1.22	1.46	−0.07	0.08	0.21	0.11	3.00
Earliglow $S_{2}$	90	*FW7*	1.80	3.38	4.29	−1.25	< 0.0001	−0.33	0.34	0.27
Earliglow $S_{1}$ and $S_{2}$	416	*FW6*	1.05	1.13	2.73	−0.84	< 0.0001	0.76	< 0.0001	0.90
		*FW7*	1.47	1.68	2.00	−0.27	0.009	0.05	0.72	0.19

^a^ The Earliglow $S_{1}$ family was developed by self‐pollinating Earliglow, an ($S_{0}$) individual heterozygous for *FW6* and *FW7*. The Earliglow $S_{2}$ family was developed by self‐pollinating a single Earliglow $S_{1}$ individual that was homozygous for *FW6* and heterozygous for *FW7*.

^b^ Statistics were estimated using Axiom 50K array SNP markers associated with the segregation of *FW6* (AX‐184674612) and *FW7* (AX‐184026067) among Earliglow $S_{1}$ and $S_{2}$ progeny. AX‐184674612 mapped to chromosome 2B, whereas AX‐18402606 mapped to chromosome 2A.

^c^ ${\bar{y}}_{--}$, ${\bar{y}}_{+-}$, and ${\bar{y}}_{++}$ are estimated marginal means (EMMs) for resistance score for *FW6*‐ and *FW7*‐associated SNP marker genotypes segregating among $S_{1}$ and $S_{2}$ progeny.

^d^ Additive and dominance effects of *FW6* and *FW7* were estimated by linear contrasts among EMMs. The additive effect ($\hat{a}$) was estimated by $\hat{a}$ = (${\bar{y}}_{++}$ ‐ ${\bar{y}}_{--}$)/2 and the dominance effect ($\hat{d}$) was estimated by $\hat{d}$ = ${\bar{y}}_{+-}$ ‐ (${\bar{y}}_{++}$ + ${\bar{y}}_{--}$)/2. Pr > *F* is the probability of a smaller *F*‐statistic by chance under the null hypothesis of no additive or dominance effect.

**TABLE 4:** Accuracy of *FW6*‐ and *FW7*‐associated single nucleotide polymorphisms (SNPs) as predictors of Fusarium wilt race 1 resistance phenotypes among Earliglow $S_{1}$ progeny.

Locus	SNP ID^a^	SNP^b^	CHR	Position (bp)^c^	PA (%)^d^	${\text{PA}}_{++}$ (%)^e^	FP (%)^f^
*FW6*	AX6	A/G	2B	1,036,551	85.0	100.0	1.2
*FW7*	AX7	A/G	2A	24,973,771	75.2	92.0	4.0
*FW6* + *FW7*	AX6 + AX7	AA/GG	2B/2A	—	92.9	100.0	4.6

^a^ AX6 is the 50K array SNP associated with *FW6* (AX‐184674612). AX7 is the 50K array SNP associated with *FW7* (AX‐184026067).

^b^ For both SNPs (A/G), A was associated with the favorable *FW6* or *FW7* allele and G was associated with the unfavorable *FW6* or *FW7* allele.

^c^ Physical position of the SNP in the Royal Royce genome.

^d^ $\text{PA}=n_{M}/n\times 100$, where $n_{M}$ is the sum of the number of $S_{1}$ individuals with A/A or A/G genotypes that predicted the resistant phenotype and G/G genotypes that predicted the susceptible phenotype and n is the number of $S_{1}$ individuals.

^e^ ${\text{PA}}_{+/+}=n_{+/+}/n_{A/A}\times 100$, where $n_{+/+}$ is the number of $S_{1}$ individuals with the A/A genotype that predicted the resistant phenotype and $n_{A/A}$ is the number of $S_{1}$ individuals with the A/A genotype.

^f^ $\text{FP}=n_{\text{FP}}/n\times 100$, where $n_{\text{FP}}$ is the number of $S_{1}$ individuals with the *A* allele that were classified as susceptible (false positives) and n is the number of $S_{1}$ individuals.

Our analyses of the $S_{2}$ family showed that the effect of the favorable *FW7* allele is nearly additive ($|\hat{d}/\hat{a}|=0.27$) when *FW6* is homozygous recessive (*fw6/fw6*) and undetectable when *FW6* is heterozygous or homozygous for the favorable allele (Figure 4; Table 3). The additive and dominance effects of *FW7* were nonsignificant in the $S_{1}$ family. Consequently, the degree‐of‐dominance estimate for *FW7* in the $S_{1}$ family was non‐informative. Although the additive effect of *FW7* was highly significant in the $S_{2}$ family (p<0.0001), the resistance of individuals homozygous for the favorable *FW7* allele and unfavorable *FW6* allele ($\bar{y}=1.80$) was weaker than that observed among individuals homozygous for the favorable *FW6* allele ($\bar{y}=1.05$). We concluded that the favorable *FW7* allele does not confer complete resistance and is not necessary for resistance in individuals carrying *FW6* or one of the other dominant *R*‐genes in the *FW1* cluster.

Using the same *FW6*‐ and *FW7*‐associated SNPs as predictors of resistance, we found that the original phenotypic assignments of $S_{1}$ individuals to resistant and susceptible classes were highly accurate (Figure 4). The percentage of $S_{1}$ individuals homozygous for susceptible alleles that were falsely declared to be resistant was 1.2% for *FW6* and 4.0% for *FW7* (Table 4). Mostly importantly from a MAS perspective, we found that AX‐184674612 and AX‐184026067 SNP genotypes predicted *FW6* homozygotes (*FW6/FW6*) and double homozygotes (*FW6/FW6 FW7/FW7*) disease resistance phenotypes with perfect accuracy (${\text{PA}}_{++}=100\%$). These DNA markers and others in LD with *FW6* and *FW7* should facilitate efficient stacking of the associated favorable alleles by MAS (Table 1).

### 3.5 *FW6* appears to be identical‐by‐descent to *FW2*

**FIGURE 5:**
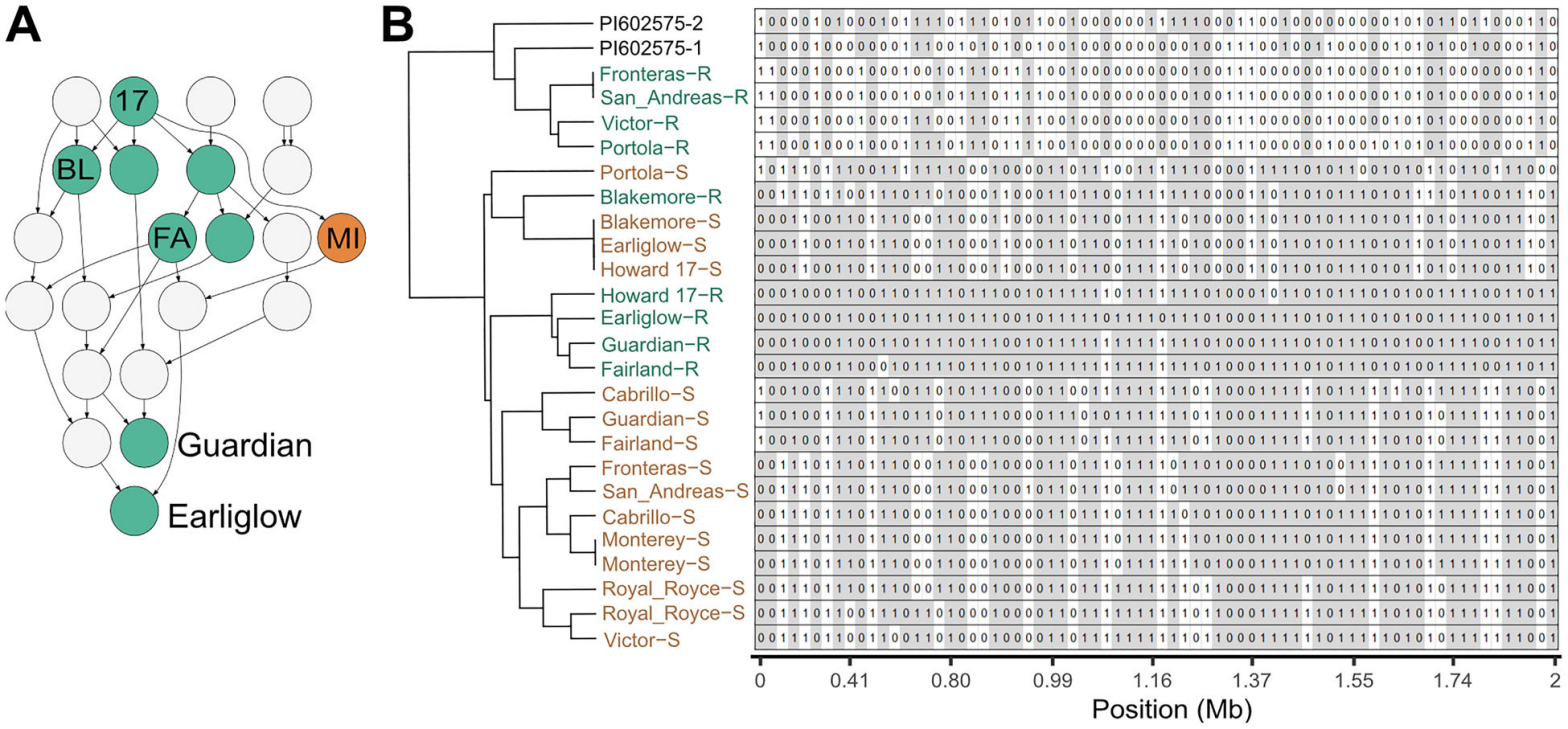
Haplotypes for 72 single nucleotide polymorphisms (SNPs) spanning 0–2 Mb on chromosome 2B among individuals known to be heterozygous for *FW1*, *FW2*, *FW5*, and *FW6* alleles. (A) Pedigrees for the cultivars Earliglow and Guardian tracing back to race 1 resistant common ancestors Howard 17 (H17), Blakemore (BL), and Fairland (FL). Nodes for individuals with known race 1 resistance phenotypes are shown in green (resistant) or orange (susceptible). The light gray nodes are individuals with unknown race 1 resistance phenotypes. Midland (MI) was the only susceptible cultivar among ancestors with known race 1 resistance phenotypes. (B) Haplotypes for 72 50K array‐genotyped SNPs spanning the genomic segment predicted to harbor *FW1*, *FW2*, *FW5*, and *FW6*. Cultivars were genotyped with the 50K Axiom SNP array. Haplotypes for 72 SNP markers associated with the underlying *R*‐gene were hierarchically clustered. Haplotypes associated with a resistant allele are shown in green with a R suffix, whereas haplotypes associated with a susceptible allele are shown in orange with an S suffix. Fronteras, Portola, Cabrillo, Victor, and San Andreas are heterozygous for *FW1*, Guardian is heterozygous for *FW2*, PI602575 is heterozygous for *FW5*. Royal Royce, Cabrillo, and Monterey are homozygous for susceptible alleles. Haplotypes associated with the resistant and susceptible *FW5* alleles could not be deduced for PI602575.

Our analyses of the breeding history of Earliglow suggested that *FW6* might be identical‐by‐descent to *FW2*, an *R*‐gene in the *FW1* cluster previously identified in the cultivar Guardian (Figure 5A; Pincot et al. 2022). These cultivars share several resistant common ancestors, including Howard 17, Blakemore, and Fairland. To investigate this, we phased 72 SNPs spanning the upper 2 Mb of chromosome 2B, assembled and clustered haplotypes, and deduced which haplotypes were associated with dominant (resistant) alleles (*FW1*, *FW2*, *FW5*, or *FW6*). The resistant individuals selected for analysis were heterozygous for *FW1* (Fronteras, San Andreas, Victor, and Portola), *FW2* (Guardian), *FW5* (PI602575), or *FW6* (Earliglow) (Figure 5). Two distinct clusters of resistant haplotypes emerged, one in individuals heterozygous for *FW1* and another in individuals heterozygous for *FW2* or *FW6* (green labeled nodes in Figure 5). The resistant *FW1* haplotypes of Fronteras and other *FW1* heterozygotes were deduced by comparing their haplotypes to haplotypes of close relatives known to be homozygous for the recessive (susceptible) allele (Cabrillo, Monterey, and Royal Royce)—resistant haplotypes are labeled with the suffix R, whereas susceptible haplotypes are labeled with the suffix S in Figure 5B. The resistant haplotypes of the four *FW1* resistant heterozygotes (Fronteras, Portola, San Andreas, and Victor) were identical in the uppermost 14‐SNP haploblock (0.0–0.6 Mb) and nearly identical across the entire 2 Mb haploblock.

The resistant haplotypes of *FW2* and *FW6* heterozygotes (Earliglow, Guardian, Fairland, and Howard 17) were deduced by comparing them to *FW1* resistant haplotypes and haplotypes observed in susceptible homozygotes and by their similarities to each other (close clustering). They were identical in the uppermost 11‐SNP haploblock (0.07–0.49 Mb), where the *FW1* cluster of *R*‐genes appears to be found and were nearly identical across the entire 2 Mb haploblock (Figures 2 and 5; Pincot et al. 2022). Although the causal genes have not yet been identified, our haplotype analysis suggests that *FW6* is identical‐by‐descent to *FW2*, and that the *R*‐gene they inherited was transmitted by Howard 17 (Figure 5). The resistant haplotype in the cultivar Blakemore, another Howard 17 descendant, appears to be novel. The Blakemore‐S haplotype clustered with other susceptible haplotypes, whereas the Blakemore‐R haplotype was dissimilar to the Earliglow‐R, Guardian‐R, and Fronteras‐R haplotypes; hence, Blakemore could be heterozygous for a novel *R*‐gene.

### 3.6 *FW7* is associated with genes predicted to encode defense‐related receptor kinases or nucleotide binding site leucine‐rich repeat proteins

We suspected that the causal gene underlying *FW7* might be mutated or not present in Royal Royce; this line is a susceptible homozygote (*fw7/fw7*), and *FW7*‐associated SNPs we identified mapped ambiguously among chromosomes 2A, 2B, 2C, and 2D in the Royal Royce genome. To overcome this, we assembled a phased Earliglow genome using HiFi‐assembled contigs scaffolded to the Royal Royce genome (FaRR1). Both Earliglow1 and Earliglow2 phases had similar assembly metrics and BUSCO scores (Supporting Information S7). Moreover, when they were aligned using GeneSpace (Lovell et al., 2022), near‐perfect synteny was observed among annotated genes identified in both Earliglow and FaRR1 across the 4 Mb haploblock predicted to harbor *FW7* (Figure S3). Using this, we identified several genes with putative defense‐related functions, in both Earliglow1 (the presumed resistant haplotype) and Royal Royce, in the LD block predicted to harbor *FW7* (Table 5; Figure 3D). We also identified 26 genes annotated in Earliglow1, but not Royal Royce and Earliglow2; however, none of these were predicted to have defense‐related functions and were therefore not considered candidates.

**TABLE 5:** Genes annotated with disease resistance functions found in the 4 Mb window predicted to harbor *FW7* on chromosome 2A.

Acronym	Position (bp)^a^	FaRR1 Gene ID^b^	EG Gene ID^c^	Putative function^d^
RK1	20,211,803–20,215,625	Fxa2Ag102878	g11072	Leucine‐rich repeat (LRR) receptor‐like Ser/Thr protein kinase
RK2	20,242,564–20,244,993	Fxa2Ag102884	g11076	G‐type lectin S‐receptor‐like Ser/Thr‐kinase
RK3	20,533,867–20,538,872	Fxa2Ag102923	g11117	G‐type lectin S‐receptor‐like Ser/Thr‐kinase
RK4	20,736,662–20,740,181	Fxa2Ag102958	g11148	Cysteine‐rich receptor‐like protein kinase
RK5	20,741,015–20,742,043	Fxa2Ag102960	g11149	Cysteine‐rich receptor‐like protein kinase
RK6	20,742,867–20,743,965	Fxa2Ag102961	g11150	Cysteine‐rich receptor‐like protein kinase
RK7	21,303,508–21,310,478	Fxa2Ag103067	g11234	LRR receptor‐like serine/threonine‐protein kinase
RK8	21,754,456–21,756,426	Fxa2Ag103144	g11307	LRR protein kinase family protein
RK9	23,537,947–23,541,218	Fxa2Ag103457	g11625	Receptor‐like serine/threonine kinase
RK10	24,068,225–24,070,889	Fxa2Ag103554	g11716	Leucine‐rich repeat protein kinase family protein
NLR1	24,227,078–24,229,327	Fxa2Ag103581	g11747	Nucleotide binding site (NBS)‐LRR
NLR2	24,234,027–24,237,955	Fxa2Ag103583	g11750	NBS‐LRR

^a^ Physical position of the gene in the Earliglow genome.

^b^ Royal Royce orthologs as identified using GENESPACE (Lovell et al., 2022).

^c^ Gene IDs from BRAKER annotation of the Earliglow genome.

^d^ Functional annotation from Royal Royce reference genome and/or protein family membership predicted by InterPro (Paysan‐Lafosse et al., 2023).

We found 1179 annotated genes in the 20–25 Mb segment on chromosome 2A predicted to harbor *FW7* (Figure 3C,D), and searched for genes with known defense‐related functions, for example, genes encoding receptor kinases (RKs) and NLR proteins (Jones & Dangl, 2006; Jacob et al., 2013; Lolle et al., 2020; Shao et al., 2016; Zhong & Cheng, 2016). Among the genes in LD with *FW7*, 10 were predicted to encode RKs and two were predicted to encode NLRs (Figure 3D; Table 5; Figure S4). NLR1, NLR2, and RK10 were most closely associated with the lead *FW7*‐associated SNP identified by QTL mapping and SNPs with the widest allele frequency differences between resistant and susceptible $S_{2}$ bulks (Figure 3; Table 1). The physical locations of NLR1 and NLR2 in particular (24,227,078–24,237,955 Mb) were proximal to the lead *FW7*‐associated SNP (AX‐184026067; 24,973,771 Mb) and flanked by the three most significant WGS‐BSA identified SNPs (Tables 1 and 5; Figure 3D). The incomplete dominance of *FW7*, however, is uncharacteristic of NLR *R*‐genes, which are typically dominant and confer complete immunity (Hammond‐Kosack & Jones, 1996; Jones & Dangl, 2006; Lolle et al., 2020).

## 4 DISCUSSION

The present study builds on earlier studies undertaken to shed light on the genetics of resistance to soilborne pathogen‐caused diseases of strawberry (Fusarium wilt, Verticillium wilt, Phytophthora crown rot, and Macrophomina) and identify elite and exotic sources of favorable alleles needed to address long‐standing and emerging disease resistance breeding problems in strawberry (Feldmann, Pincot, Vachev et al., 2024; Jiménez et al., 2023; Knapp et al., 2024; Pincot et al., 2018, 2020, 2022). Among those four diseases, monogenic (gene‐for‐gene) resistance has only been reported for Fusarium wilt, specifically for *Fof* races found in California and Japan (Henry et al., 2021; Mori et al., 2005; Pincot et al., 2018, 2022). The resistance previously reported for a race found in Australia was polygenic (Paynter et al., 2014).

We initially screened diverse genetic resources for resistance to California race 1 of the Fusarium wilt pathogen because nothing was known about the genetics of resistance and we suspected that favorable alleles were either rare or absent in modern cultivars and highly dispersed; a prediction supported by the discovery of *FW3* and *FW4* in a previous study and *FW7* in the present study (Figure 3; Pincot et al. 2022). Earliglow was one of the more intriguing sources of resistance to emerge from our original screening studies, partly because resistance to California race 1 appeared to be caused by unlinked dominant duplicate epistasis and partly because this cultivar was one of only three that were found to be resistant to races of the pathogen found in Australia and Japan where the disease was first reported in strawberry (Henry et al., 2021; Mori et al., 2005; Okamoto et al., 1970; Pincot et al., 2022; Winks & Williams, 1965). Under restrictive screening conditions in a completely enclosed growth chamber, Henry et al. (2021) previously found Earliglow to be resistant to virulent *Fof* races found in Australia and Japan—strict quarantine screening procedures were necessary because those *Fof* races were either not present or have not yet been discovered in California. Our findings suggest that Earliglow might be a source of monogenic resistance to *Fof* races found in Australia and Japan, but that still needs to be tested under greenhouse or field screening conditions less restrictive than those that were necessary for pathogen containment in our previous studies (Henry et al., 2021).

The discovery of *FW7* increases the number of independent Fusarium wilt race 1 resistance loci to four in strawberry, at least one of which appears to be a cluster of duplicated *R*‐genes that includes *FW1*, *FW2*, *FW5*, and *FW6* (Hammond‐Kosack & Jones, 1997; Jones & Dangl, 2006; Meyers et al., 2003; Michelmore & Meyers, 1998). The *R*‐genes underlying the four loci were discovered in diverse genetic resources, none of which are known to have coevolved with virulent races of the pathogen—Fusarium wilt has not been reported in any of the North American locations, outside of California, where resistant genetic resources originated or where natural populations found in gene banks were collected (Koike et al., 2009; Koike & Gordon, 2015; Pincot et al., 2022). The spatial distribution of virulent *Fof* races worldwide, dynamics of pathogen spread, and host–pathogen coevolution are not completely clear (Dilla‐Ermita et al., 2023; Henry et al., 2017). Have cryptic *Fof* infections by the pathogen been widespread (Stergiopoulos & Gordon, 2014)? Has the pathogen asymptomatically spread over long distances? There are many unanswered questions about the landscape epidemiology of *Fof*, a pathogen that typically spreads through infested soils and infected plants (Gordon, 2017; Henry et al., 2017). Importantly, Henry et al. (2023) showed that *Fof* could spread through airborne conidia, a factor that complicates management of the disease and presumably promotes the spread of the pathogen.

The diversity and dispersion of favorable alleles for resistance to Fusarium wilt in strawberry have largely been driven by the formation of populations that arise naturally along the path of domestication and of course by the evolution of *R*‐genes in natural populations (Gaut et al., 2015; Jones & Dangl, 2006; Meyers et al., 2003; Michelmore & Meyers, 1998; Miller & Gross, 2011; Pincot et al., 2022; Purugganan & Fuller, 2009). The Fusarium wilt *R*‐genes found in domesticated populations and individuals like Earliglow have been determined by their fortuitous presence in founders upstream of breeding bottlenecks, their survival in bottlenecked populations through random genetic drift and natural selection, and the absence of artificial selection pressure before 2016 (Hardigan, Lorant, et al., 2021; Pincot et al., 2018, 2022). We obviously cannot rule out natural selection pressure, cryptic pathogen presence, or latent infections (Gordon, 2017; Stergiopoulos & Gordon, 2014). The founders of the elite population we studied (the California population) were selected long before Fusarium wilt emerged as a disease problem in California, gene‐for‐gene resistance to the pathogen was discovered, and MAS strategies for pyramiding *R*‐alleles could be envisioned (Feldmann, Pincot, Seymour et al., 2024; Hardigan, Lorant, et al., 2021; Pincot et al., 2018, 2022). The development of Fusarium wilt resistant cultivars for California was not necessary until the pathogen suddenly emerged in 2005, did not become a breeding priority until disease surveys later showed that the pathogen was causing widespread plant death, and was not initiated until our breeding and genetic studies got underway in 2015 (Henry et al., 2017, 2019, 2023; Koike & Gordon, 2015; Pincot et al., 2018). As a consequence, the frequency of the favorable *FW1* allele, which drifted and fortuitously survived breeding bottlenecks, was only 0.16 in the elite genetic resources screened in our original study (Pincot et al., 2022). That allele has since been driven to near fixation by phenotypic and MAS and deployed in short‐day, day‐neutral, and summer‐plant cultivars (Cole, Knapp, et al., 2025; Cole, Pincot, et al., 2025; Knapp et al., 2023, 2025).

Our search for Fusarium wilt resistance loci and alleles beyond *FW1* was driven by the concern that resistance to California race 1 might not be durable, other races of the pathogen might be present but undiscovered in California, *R*‐genes effective against those races might not exist or could be exceedingly rare, and resistance to additional races would likely be needed. Indeed, California race 2 was not discovered until race 1 resistant cultivars carrying *FW1* were deployed (Dilla‐Ermita et al., 2023). The Fusarium wilt resistance loci discovered on chromosomes 1A, 2A, 2B, and 6A are likely sources of favorable alleles for resistance to other virulent races of the pathogen (Figures 2 and 3; Pincot et al. 2022), although much is unknown about the genetics of resistance to races found outside of California (Henry et al., 2021; Mori et al., 2005; Paynter et al., 2014). The pathogen races studied to date appear to vary in host differential response, pathogenicity, and symptomatology; hence, the genetic mechanisms underlying resistance could differ and could be quantitative for some races of the pathogen (Paynter et al., 2014, 2016).

The epistatic masking of *FW7* by *FW6* among Earliglow $S_{1}$ progeny suggested that favorable alleles could be hidden in genetic resources carrying dominant, race‐specific *R*‐genes (Figure 4). This challenge commonly arises in plant breeding when resistance is conferred by a combination of qualitative and quantitative *R*‐genes, and the genetic effects of the latter are murky or masked by the former (Nelson et al., 2018; Poland et al., 2009; Poland & Rutkoski, 2016; Rutkoski et al., 2015). The *R*‐gene discovery problem described here is somewhat analogous to that encountered in breeding for quantitative resistance to leaf, stem, and stripe rust in allo‐hexaploid wheat (*Triticum aestivum*), where *R*‐genes with large effects obscure the effects of genes with small effects (Daetwyler et al., 2014; Juliana et al., 2017; Rutkoski et al., 2011). *FW3*, *FW4*, and *FW7* were discovered because the exotic donors were homozygous for susceptible alleles elsewhere in the genome (Pincot et al., 2022) or because the Mendelian distribution suggested that the resistance phenotypes were caused by dominant duplicate epistasis (Figures 2 and 3). The synergistic effects of independent Fusarium wilt resistance loci on durability are unknown in strawberry, primarily because individual and pyramided combinations of favorable *R*‐alleles need to be developed and tested head‐to‐head over a geographically wide area and over a long time period to quantify differences in the durability of resistance (Figure 2, 3, 4; Pincot et al. 2022). *FW3*, *FW4*, and *FW7* are unlinked with each other and the *R*‐genes found on chromosome 2B (*FW1*, *FW2*, *FW5*, and *FW6*) and thus could be pyramided by MAS. Although they had a weaker effect on resistance, they could provide increased protection to race 1 and other races as they emerge. The problem of pyramiding *R*‐genes masked by epistasis can be easily solved by applying MAS to linked genetic variants identified in our studies (Figures 2 and 3; Table 1; Pincot et al. 2022).

Substantiating the effects of *R*‐gene pyramids on the durability of resistance has been technically challenging in agriculturally important plants, despite the theory and logic behind them (Cowger & Brown, 2019; Mundt, 2014, 2018; Nelson et al., 2018; Poland et al., 2009; Poland & Rutkoski, 2016). The merit of pyramiding Fusarium wilt *R*‐genes in strawberry is unclear. We assume that pyramiding independent *R*‐genes could be an effective strategy for increasing the durability of resistance because the molecular mechanisms and host–pathogen interactions underlying them presumably differ and decrease the vulnerability of the host to pathogen evolution (Dilla‐Ermita et al., 2023; Henry et al., 2017, 2021; Mundt, 2014, 2018; Nelson et al., 2018). However, real‐world examples of *R*‐gene pyramids are limited because of the technical difficulty of deploying and comparing them over time and space, and because the host–pathogen interactions are exceedingly complex and difficult to predict (Juliana et al., 2017; Mundt, 2014, 2018; Rutkoski et al., 2011, 2015).

When Pincot et al. (2018) discovered the *FW1* *R*‐gene, the only *Fof* race discovered in California was race 1 (Henry et al., 2017). Cultivars known to carry *FW1*, a race 1 *R*‐gene, became sentinels for discovering previously unknown *Fof* races in California. Shortly after we reported the discovery of several novel California race 1 *R*‐genes (*FW2*–*FW5*; Pincot et al. 2022), a report surfaced that race 1 resistant cultivars were succumbing to *Fof* infections in organic production fields in southern California (Dilla‐Ermita et al., 2023). Subsequent analyses showed that those plants were infected by a unique and previously unidentified *Fof* race (California race 2) that appeared to be geographically limited (Dilla‐Ermita et al., 2024). Hence, the widespread deployment of race 1 resistant cultivars carrying the *FW1* gene uncovered the presence of a previously unknown *Fof* race in California. That discovery would have been difficult without the identification of host differentials and knowledge of the race specificity of the *R*‐genes that they carry (Henry et al., 2021; Pincot et al., 2018, 2022).

California race 2 could have been present in California soils long before race 1 resistant cultivars exerted the selection pressure needed to drive the evolution of the pathogen (Dilla‐Ermita et al., 2023; Gordon, 2017; Hammond‐Kosack & Jones, 1996, 1997; Jones & Dangl, 2006). We suspect that race 2 simply went undetected until race 1 resistant cultivars carrying *FW1* were grown in race 2 infested fields (Dilla‐Ermita et al., 2023; Pincot et al., 2018). That aside, the discovery of race 2 has necessitated an aggressive search for sources of resistance to complement those identified for race 1. We do not yet know if any of the Fusarium wilt resistance loci identified in our studies harbor alleles that confer resistance to *Fof* race 2 or other *Fof* races found around the world (Dilla‐Ermita et al., 2023; Henry et al., 2021). Those loci, however, supply the framework for exploring that question and expanding our understanding of natural genetic resistance to Fusarium wilt in strawberry.

## AUTHOR CONTRIBUTIONS

**Mishi V. Vachev**: Conceptualization; data curation; formal analysis; methodology; validation; visualization; writing—original draft; writing—review and editing. **Marta Bjornson**: Conceptualization; data curation; formal analysis; funding acquisition; project administration; resources; supervision; visualization; writing—original draft; writing—review and editing. **Dominique D. A. Pincot**: Conceptualization; data curation; formal analysis; investigation; visualization; writing—review and editing. **Randi A. Famula**: Data curation; investigation; project administration; resources; writing—review and editing. **John T. Lovell**: Data curation; formal analysis; writing—review and editing. **Avril M. Harder**: Data curation; formal analysis; writing—review and editing. **Lori H. Handley**: Data curation; writing—review and editing. **Jane Grimwood**: Data curation; project administration. **Hillel Brukental**: Formal analysis; writing—review and editing. **Cindy M. Lopez**: Writing—review and editing. **Glenn S. Cole**: Conceptualization; investigation; project administration; resources; writing—review and editing. **Mitchell J. Feldmann**: Data curation; formal analysis; funding acquisition; investigation; project administration; supervision; validation; visualization; writing—original draft; writing—review and editing. **Steven J. Knapp**: Conceptualization; formal analysis; funding acquisition; investigation; project administration; resources; visualization; writing—original draft; writing—review and editing.

## CONFLICT OF INTEREST STATEMENT

The authors declare no conflicts of interest.

## Supporting information




**Supplemental File S1**. Fusarium wilt race 1 resistance scores for 350 ‘Earliglow’ $S_{1}$ n=350 individuals recorded on seven different dates six to 12 weeks post‐inoculation.
**Supplemental File S2**. 50K array SNP genotypes (k=49,483) for an ‘Earliglow’ $S_{1}$ family (n=327).
**Supplemental File S3**. Fusarium wilt race 1 resistance scores for 109 ‘Earliglow’ $S_{2}$ individuals recorded on four different dates six to 12 weeks post‐inoculation.
**Supplemental File S4**. Genotypic data for ‘Earliglow’ $S_{2}$ n=90 progeny and m=8,925 variants after filtering from the 50K SNP array.
**Supplemental File S5**. Genotypic data for ‘Earliglow’ $S_{2}$ progeny from the bulked‐segregant analysis in VCF format including data from four bulks with m=5,279,933 variants.
**Supplemental File S6**. Allele‐specific forward and common reverse primer sequences for KASP markers targeting SNPs associated with the *FW6* locus on chromosome 2B. The physical positions reported for these SNPs are in the ‘Royal Royce’ genome.
**Supplemental File S7**. Genome assembly statistics for ‘Royal Royce’, ‘Earliglow1’, and ‘Earliglow2’ genomes.
**Supplemental Fig. S1**. Number of genetic variants discovered between DNA sequence bulks of Fusarium wilt resistant and susceptible $S_{2}$ individuals across the ‘Earliglow’ genome.
**Supplemental Fig. S2**. Allele frequency differences for genetic variants identified between DNA sequence bulks of Fusarium wilt resistant and susceptible $S_{2}$ individuals across the ‘Earliglow’ genome.
**Supplemental Fig. S3**. Synteny among genes in ‘Royal Royce’, ‘Earliglow‐R’, and ‘Earliglow‐S’ genomic segments spanning the *FW7* locus. ‘Royal Royce’ gene identifiers are shown for the 12 candidate disease resistance genes documented in Table 5.
**Supplemental Fig. S4**. Predicted protein domains of *FW7* candidate genes.

## Data Availability

Whole genome sequencing (WGS) data (raw reads) have been deposited at the NCBI Sequence Read Archive (SRA) under Bioproject #PRJNA1251051, samples: SRR33145761, SRR33145762, SRR33145763, SRR33145764, SRR33145765, SRR33145766, SRR33145767, SRR33145768, and SRR33145769. Earliglow haplotypes were submitted to NCBI Genome Database under BioProject numbers PRJNA1253725 and PRJNA1253726. The raw genotypic and phenotypic data are publicly available and have been deposited in Zenodo (https://doi.org/10.5281/zenodo.15238483).
