# Supplementary material for: Whole‐genome sequencing bulked segregant analysis uncovered FW7, a Fusarium wilt resistance gene masked by epistasis in octoploid strawberry
Source: Plant Genome. 2025 Oct 22;18(4):e70136. doi: 10.1002/tpg2.70136 (PMC12541573; doi:10.1002/tpg2.70136)
Supplement: Supplementary file 1 — Supplemental File S1. Fusarium wilt race 1 resistance scores for 350 ‘Earliglow’ S1 n=350 individuals recorded on seven different dates six to 12 weeks post‐inoculation. Supplemental File S2. 50K array SNP genotypes (k=49,483) for an ‘Earliglow’ S1 family (n=327). Supplemental File S3. Fusarium wilt race 1 resistance scores for 109 ‘Earliglow’ S2 individuals recorded on four different dates six to 12 weeks post‐inoculation. Supplemental File S4. Genotypic data for ‘Earliglow’ S2 n=90 progeny and m=8,925 variants after filtering from the 50K SNP array. Supplemental File S5. Genotypic data for ‘Earliglow’ S2 progeny from the bulked‐segregant analysis in VCF format including data from four bulks with m=5,279,933 variants. Supplemental File S6. Allele‐specific forward and common reverse primer sequences for KASP markers targeting SNPs associated with the FW6 locus on chromosome 2B. The physical positions reported for these SNPs are in the ‘Royal Royce’ genome. Supplemental File S7. Genome assembly statistics for ‘Royal Royce’, ‘Earliglow1’, and ‘Earliglow2’ genomes. Supplemental Fig. S1. Number of genetic variants discovered between DNA sequence bulks of Fusarium wilt resistant and susceptible S2 individuals across the ‘Earliglow’ genome. Supplemental Fig. S2. Allele frequency differences for genetic variants identified between DNA sequence bulks of Fusarium wilt resistant and susceptible S2 individuals across the ‘Earliglow’ genome. Supplemental Fig. S3. Synteny among genes in ‘Royal Royce’, ‘Earliglow‐R’, and ‘Earliglow‐S’ genomic segments spanning the FW7 locus. ‘Royal Royce’ gene identifiers are shown for the 12 candidate disease resistance genes documented in Table 5. Supplemental Fig. S4. Predicted protein domains of FW7 candidate genes. [file TPG2-18-e70136-s001.zip › Supplementary data/Supplemental figures/Supplemental Figure S3 Synteny FW7 candidate genes.pdf]

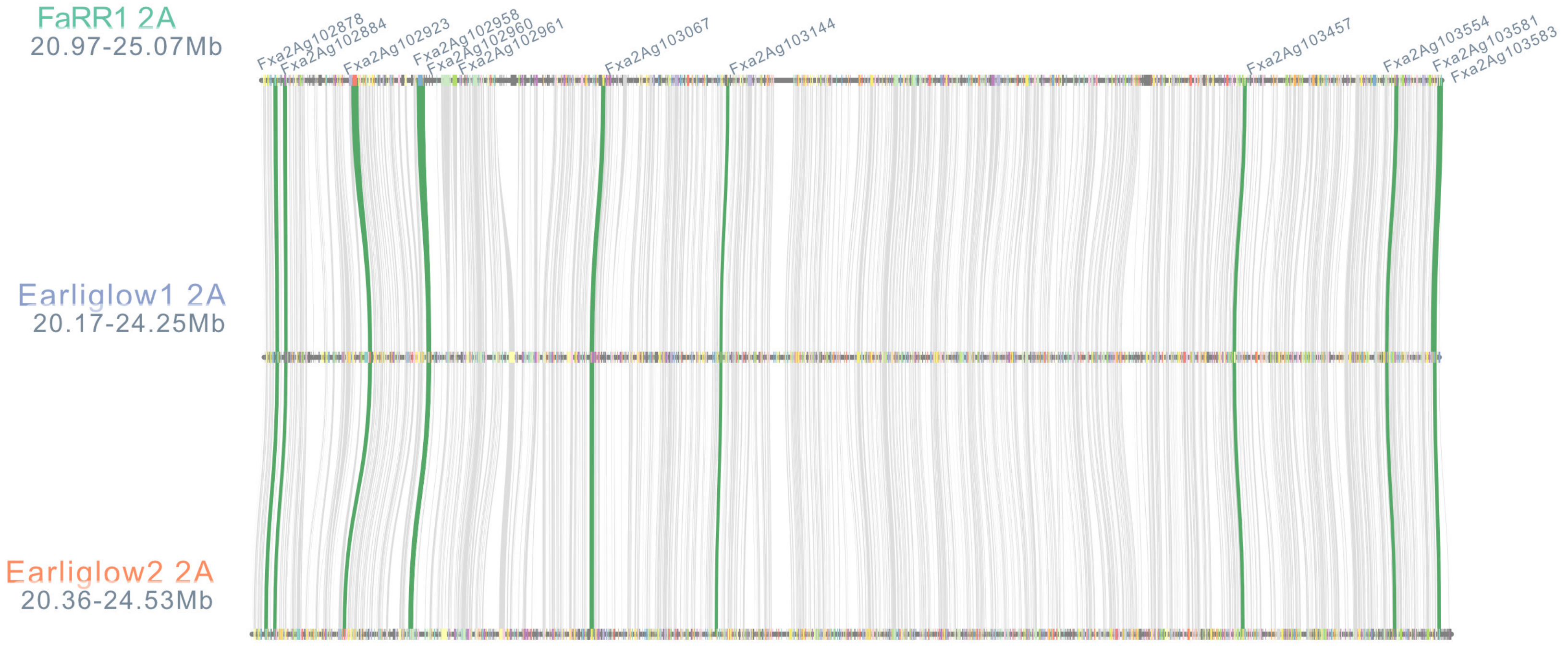
FaRR1 2A
20.97-25.07Mb
Earliglow1 2A
20.17-24.25Mb
Earliglow2 2A
20.36-24.53Mb
Fxa2Ag102878
Fxa2Ag102884
Fxa2Ag102923
Fxa2Ag102958
Fxa2Ag102960
Fxa2Ag102961
Fxa2Ag103067
Fxa2Ag103144
Fxa2Ag103457
Fxa2Ag103554
Fxa2Ag103581
Fxa2Ag103583
